# Supplementary material for: The genomic landscape of reference genomes of cultivated human gut bacteria
Source: Nat Commun. 2023 Mar 25;14:1663. doi: 10.1038/s41467-023-37396-x (PMC10039858; doi:10.1038/s41467-023-37396-x)
Supplement: Supplementary file 1 — Supplementary information [file 41467_2023_37396_MOESM1_ESM.pdf]

Supplementary information：

The genomic landscape of reference genomes of cultivated human gut bacteria

Xiaoqian Lin[1, 2&], Tongyuan Hu[1&], Jianwei Chen[1, 3, 4], Hewei Liang[1], Jianwei Zhou[3], Zhinan Wu[1, 5], Chen Ye[1], Xin Jin[1], Xun Xu[1], Wenwei Zhang[1], Xiaohuan Jing[6], Tao Yang[6], Jian Wang[1, 7], Huanming Yang[1, 7], Karsten Kristiansen[1, 3, 4, 8*], Liang Xiao[1, 3, 9*], Yuanqiang Zou[1, 3, 4, 9*]

[1] BGI-Shenzhen, Shenzhen 518083, China

[2] School of Bioscience and Biotechnology, South China University of Technology, Guangzhou 510006, China

[3] Qingdao-Europe Advanced Institute for Life Sciences, BGI-Shenzhen, Qingdao 266555, China

[4] Laboratory of Genomics and Molecular Biomedicine, Department of Biology, University of Copenhagen, Universitetsparken 13, 2100 Copenhagen, Denmark

[5] College of Life Sciences, University of Chinese Academy of Sciences, Beijing 100049， China

[6] China National GeneBank, BGI-Shenzhen, Shenzhen 518120, China

[7] James D. Watson Institute of Genome Sciences, Hangzhou 310058, China

[8] PREDICT, Center for Molecular Prediction of Inflammatory Bowel Disease, Faculty of Medicine, Aalborg University, 2450 Copenhagen, Denmark

[9] Shenzhen Engineering Laboratory of Detection and Intervention of human intestinal microbiome, BGI-Shenzhen, Shenzhen, China

[&]These authors contributed equally: Xiaoqian Lin, Tongyuan Hu.

[*]**Corresponding authors**:

Liang Xiao.

Address: BGI-Shenzhen, Beishan Industrial Zone, Shenzhen 518083, China.

E-mail: xiaoliang@genomics.cn

Karsten Kristiansen

Address: Laboratory of Genomics and Molecular Biomedicine, Department of Biology, University of Copenhagen, Universitetsparken 13, 2100 Copenhagen, Denmark.

E-mail: kk@bio.ku.dk

Yuanqiang Zou.

Address: BGI-Shenzhen, Beishan Industrial Zone, Shenzhen 518083, China.

E-mail: zouyuanqiang@genomics.cn

Supplementary Figures

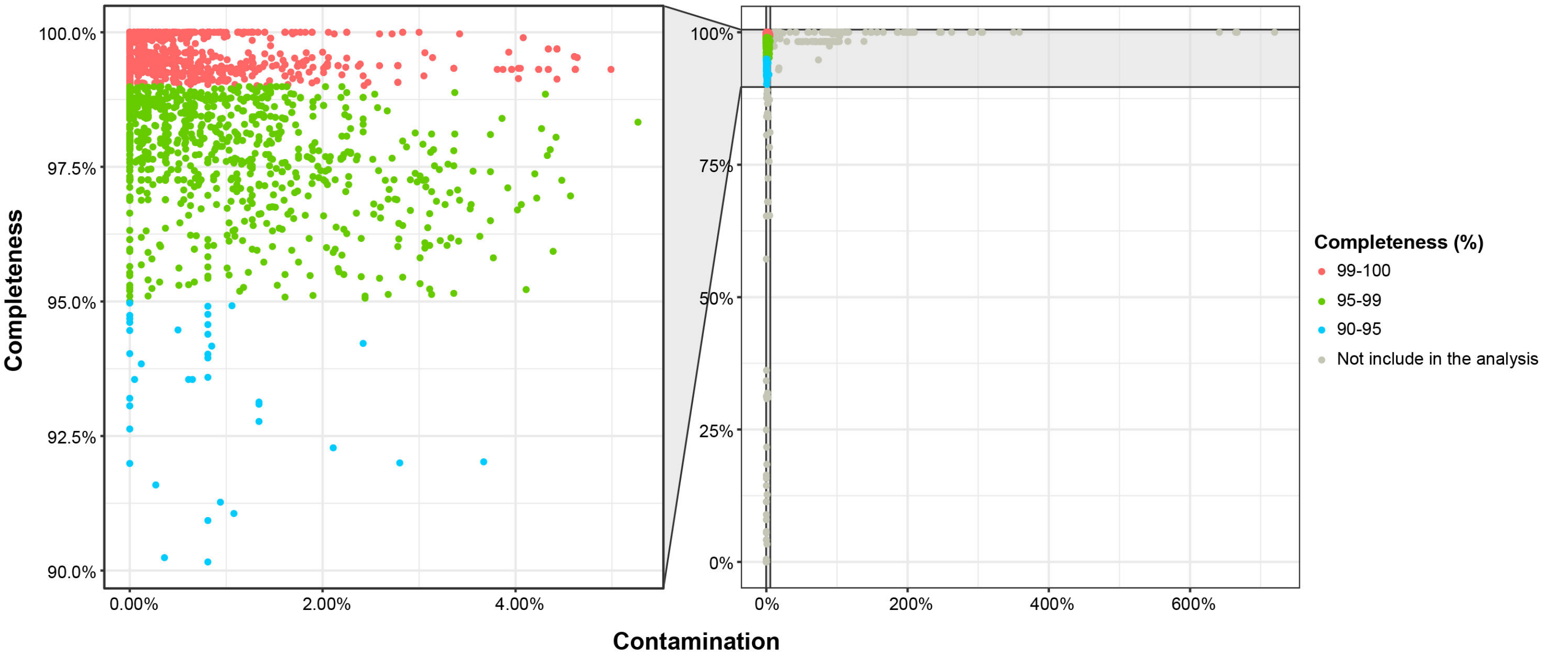

Supplementary Fig. 1: CheckM assessment of the 4,066 genomes. The genomes finally included in the CGR2 are marked as blue, green and red according to their completeness.

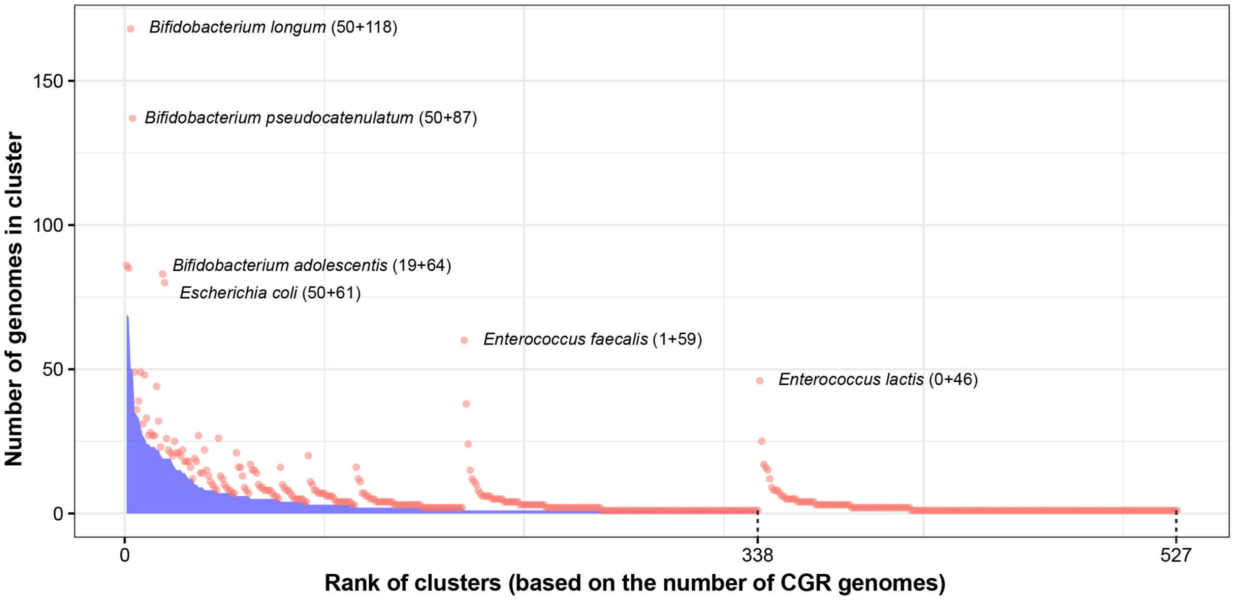

Supplementary Fig. 2: Contribution of newly sequenced genomes to the number of genomes in each cluster. Clusters were sorted based on the number of CGR genomes, blue bars represent the distribution of CGR genomes in each cluster, and red dots represent the distribution of CGR2 genomes in each cluster.

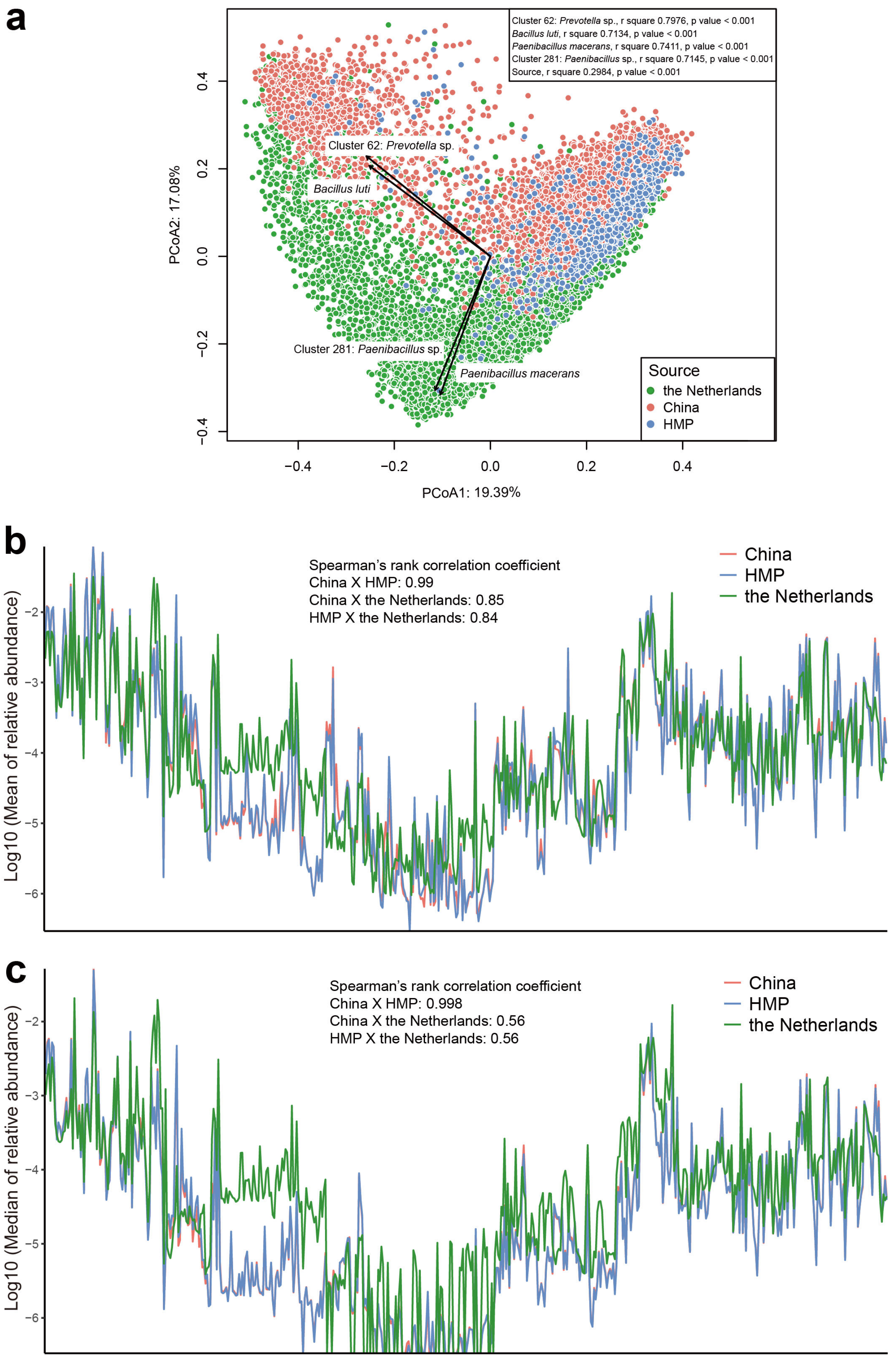

Supplementary Fig. 3: Beta diversity and relative abundance of 527 representative clusters in cohorts of China, HMP, and the Netherlands. a, Beta diversity analysis and the fitting of factors onto the ordination of bacterial community. (**b-c**), Correlation of mean (**b**) and median (**c**) of relative abundance of 527 representative clusters in healthy cohorts from China and the Netherlands, and in the HMP cohort. The x-axis represents 527 representative clusters in the same order as in Figure 1b (from top to bottom)

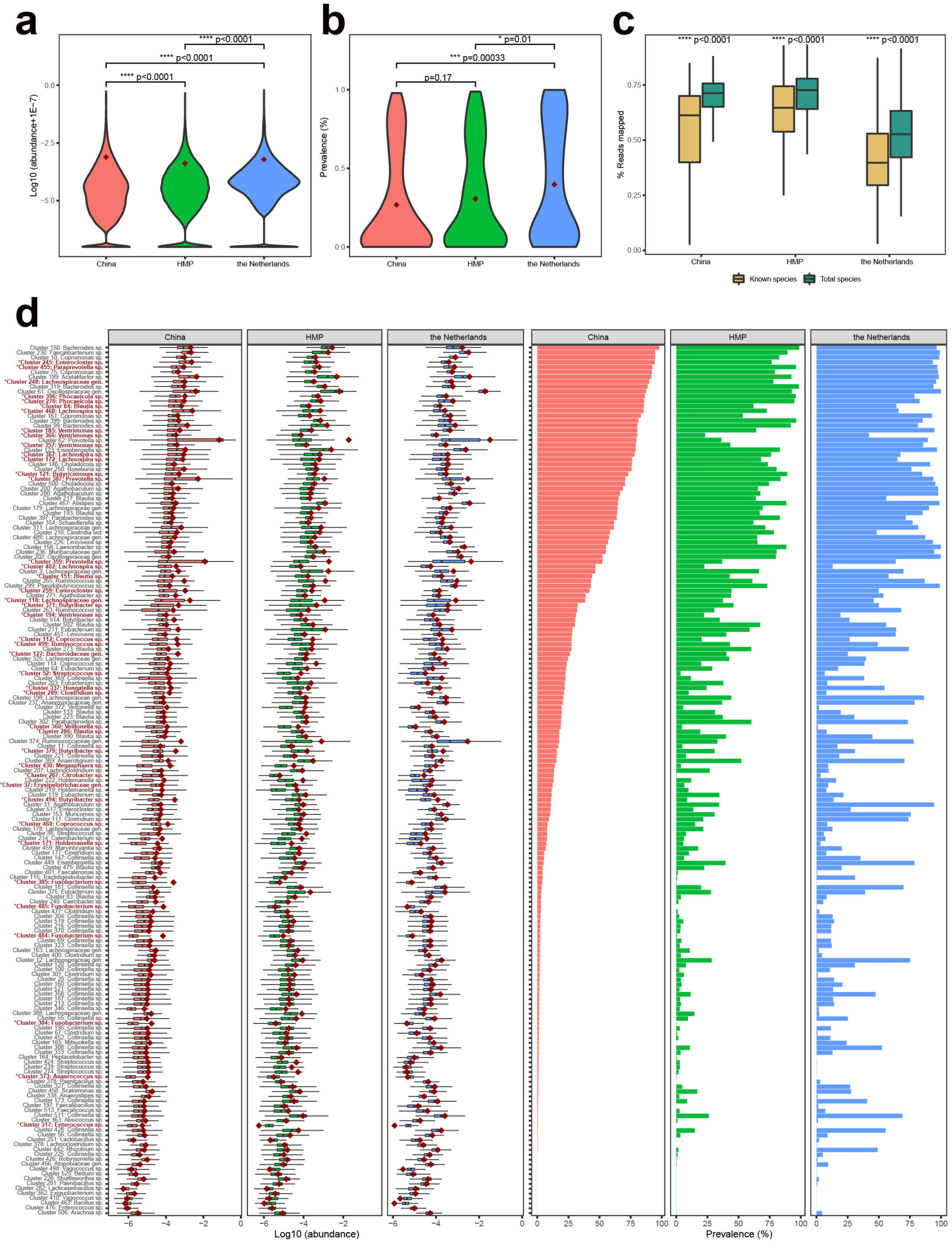

Supplementary Fig. 4: Relative abundance and prevalence of 179 previously unidentified species in healthy cohorts of China, HMP, and the Netherlands. (a-b), Comparison of relative abundance (**a**) and prevalence (**b**) between 3 cohorts. *P* values were calculated using Wilcoxon rank-sum test (two-sided) (China: *n*=635,450, HMP: *n*=118,319, the Netherlands: *n*=1,475,676, *n* values refer to the number of independent results used to derive statistics). **c**, Improvement of metagenomic

reads mapping before and after adding the 179 previously unidentified species. *P* values were calculated using Wilcoxon rank-sum test (two-sided) (China: *n*=3,550, HMP: *n*=661, the Netherlands: *n*=6,366, *n* values refer to the number of independent results used to derive statistics). **d**, Relative abundance and prevalence of each 179 previously unidentified species. Boxplots show median, 25th and 75th percentile, the whiskers indicate the minima and maxima, and the dark red points represent the mean before log transformation. Red marked species are significantly enriched in China (two-sided Wilcoxon rank-sum test, see Supplementary Table 3 for exact *P* values).

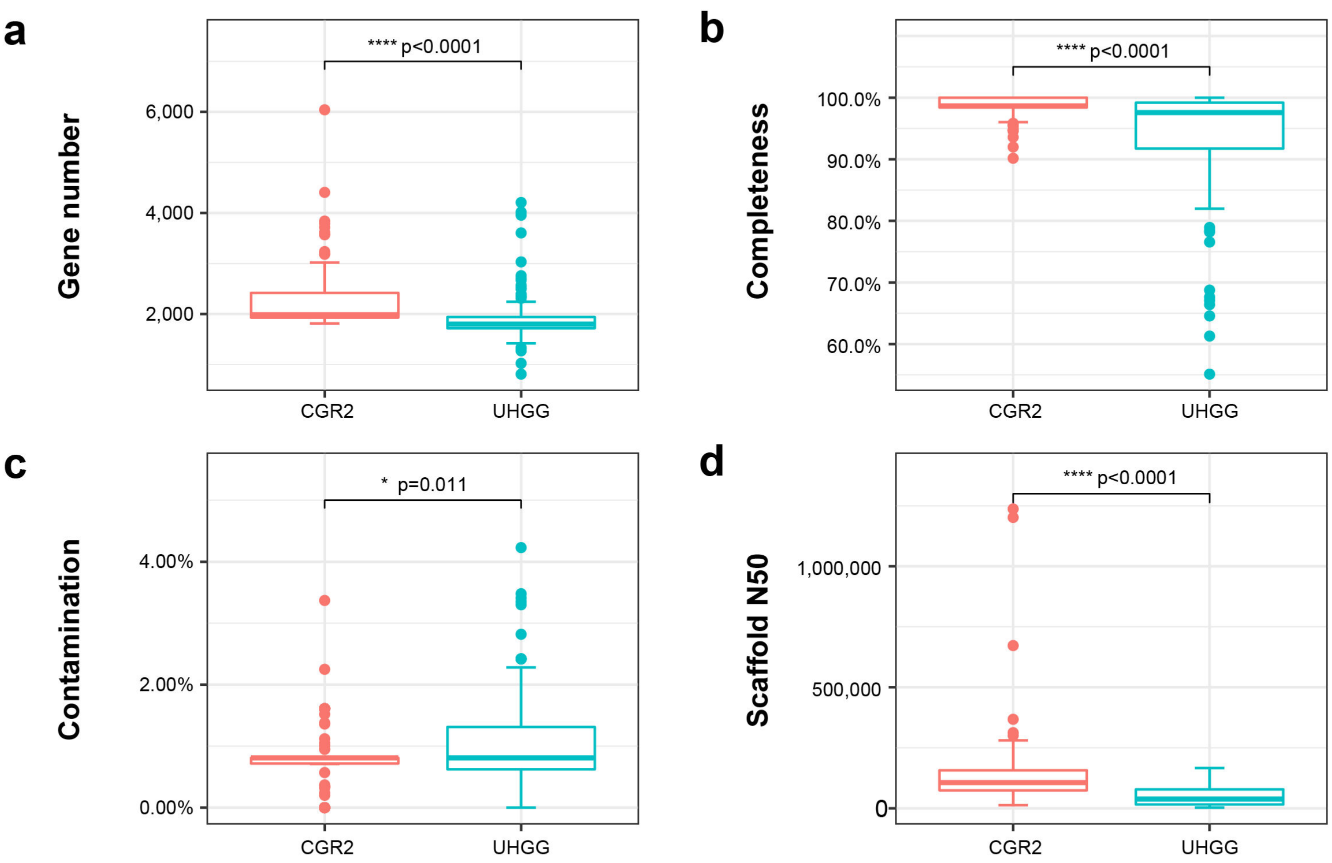

Supplementary Fig. 5: Comparison of genome information between CGR2 genomes and matched uncultured UHGG genomes. (**a-d**) Comparison of the gene number (**a**), completeness (**b**), contamination (**c**) and scaffold N50 (**d**) in the mapped genomes between CGR2 and UHGG-Unculture. Boxplots show median, 25th and 75th percentile, the whiskers indicate the minima and maxima, and the points laying outside the whiskers of boxplots represent the outliers. *P* values were calculated using Wilcoxon rank-sum test (two-sided) (CGR2: *n*=146, UHGG: *n*=126, *n* values refer to the number of independent results used to derive statistics).

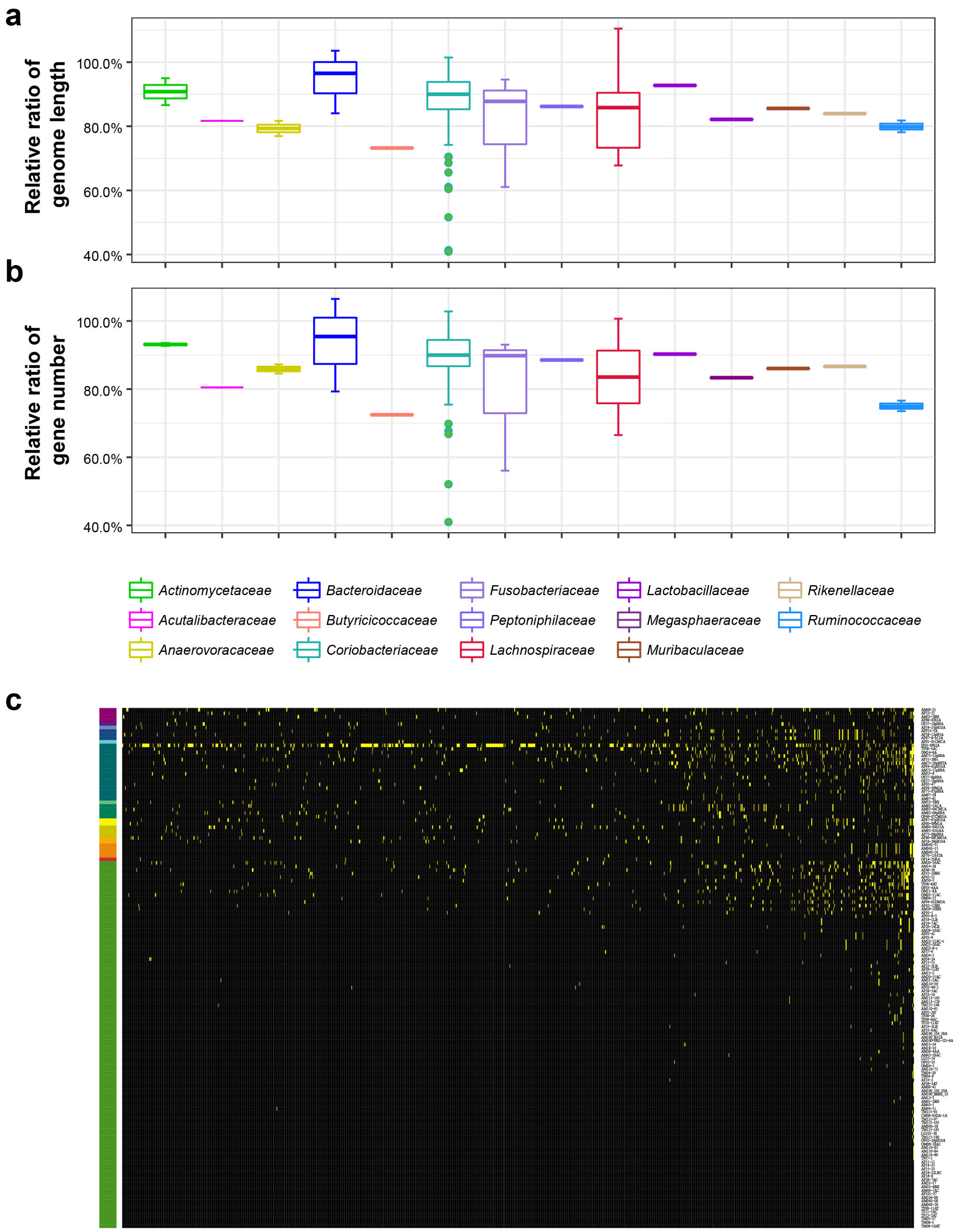

Supplementary Fig. 6: The contribution of isolate-based genomes relative to MAGs at the family level. (**a-b**) The ratio of the genome length (**a**) and gene number (**b**) of the UHGG-Uncultured relative to CGR2 in the mapped genome based on family level. Boxplots show median, 25th and 75th percentile, the whiskers indicate the minima and maxima, and the points laying outside the whiskers of boxplots represent the outliers. Different color indicates different family (*Actinomycetaceae*: *n*=2, *Acutalibacteraceae*: *n*=1, *Anaerovoracaceae*: *n*=2, *Bacteroidaceae*: *n*=3, *Butyricicoccaceae*: *n*=1, *Coriobacteriaceae*: *n*=93, *Fusobacteriaceae*: *n*=3, *Helcococcaceae*: *n*=1, *Lachnospiraceae*: *n*=13, *Lactobacillaceae*: *n*=1, *Megasphaeraceae*: *n*=1, *Muribaculaceae*: *n*=1, *Rikenellaceae*: *n*=1, *Ruminococcaceae*: *n*=3, *n* values refer to the number of independent genomes results used to derive statistics). **c,** The distribution of unique genes of isolate genome relative to MAGs.

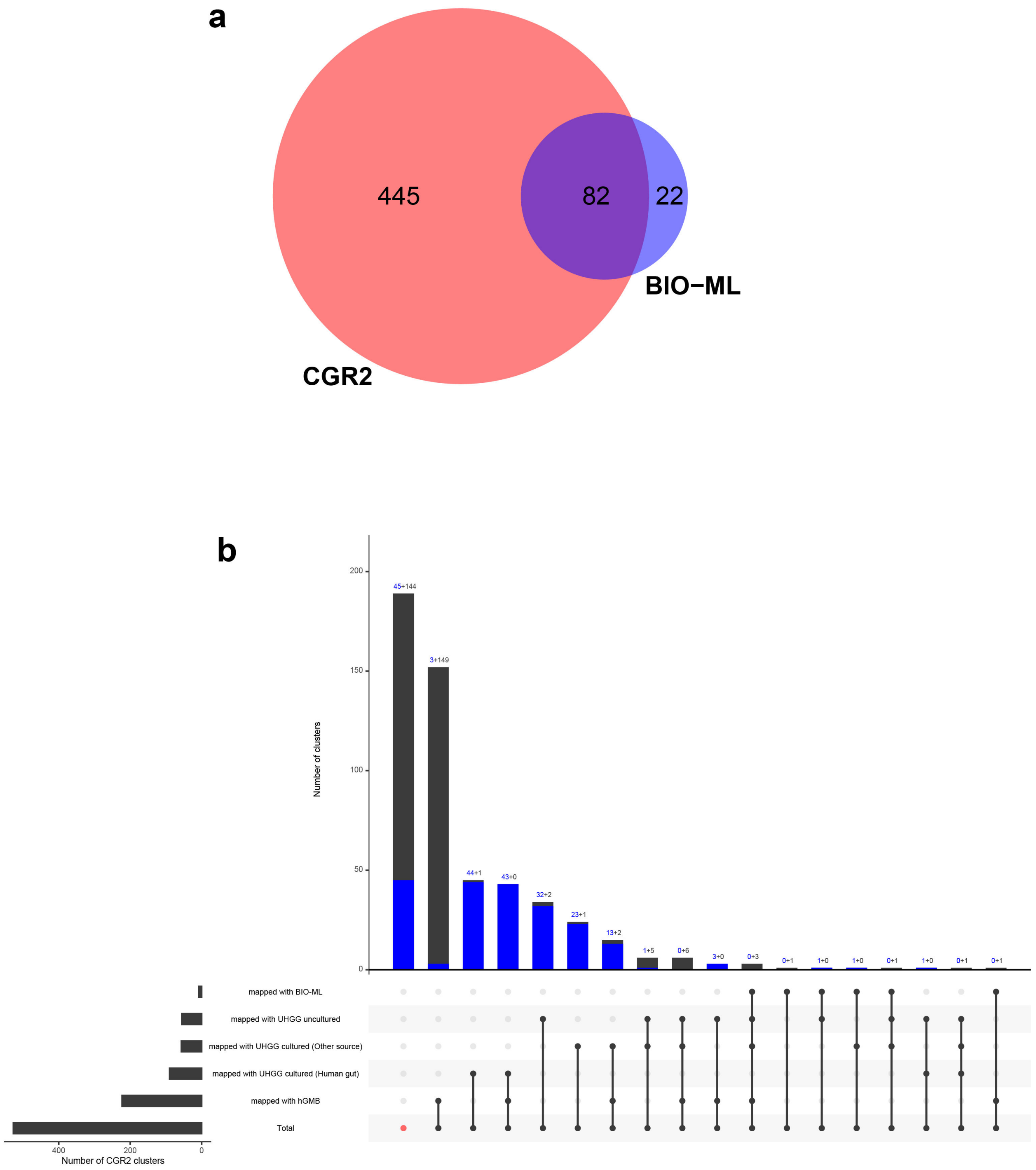

Supplementary Fig. 7: Mapping of CGR2 genomes against existing collections. a, Venn diagram illustrating the comparison between the CGR2 species-level clusters and the BIO-ML species-level clusters. **b**, Overall comparison of CGR2 with existing datasets. The number of CGR2 clusters unique to CGR is colored in blue.

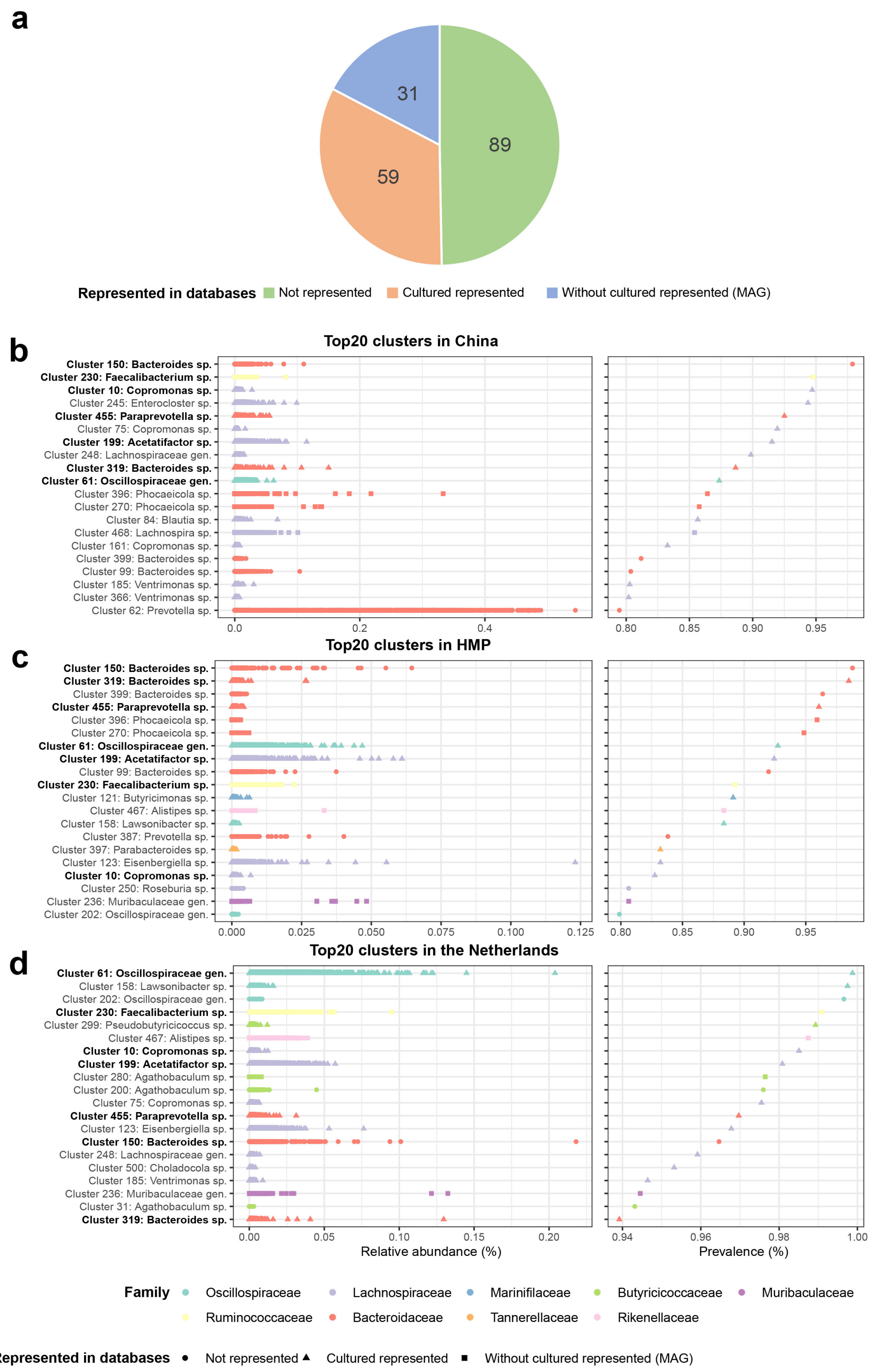

Supplementary Fig. 8: Representativeness of 179 previously unidentified species across the databases. a, Presence of 179 previously unidentified species in published cultured databases (cultured genomes of UHGG, BIO-ML, and hGMB) and MAG databases (uncultured genomes of UHGG). **b-d,** The prevalence of top 20 previously unidentified species in China **(b)**, HMP **(c)** and Netherlands **(d)**. Colors represent family, and shapes represent occurrences in the databases.

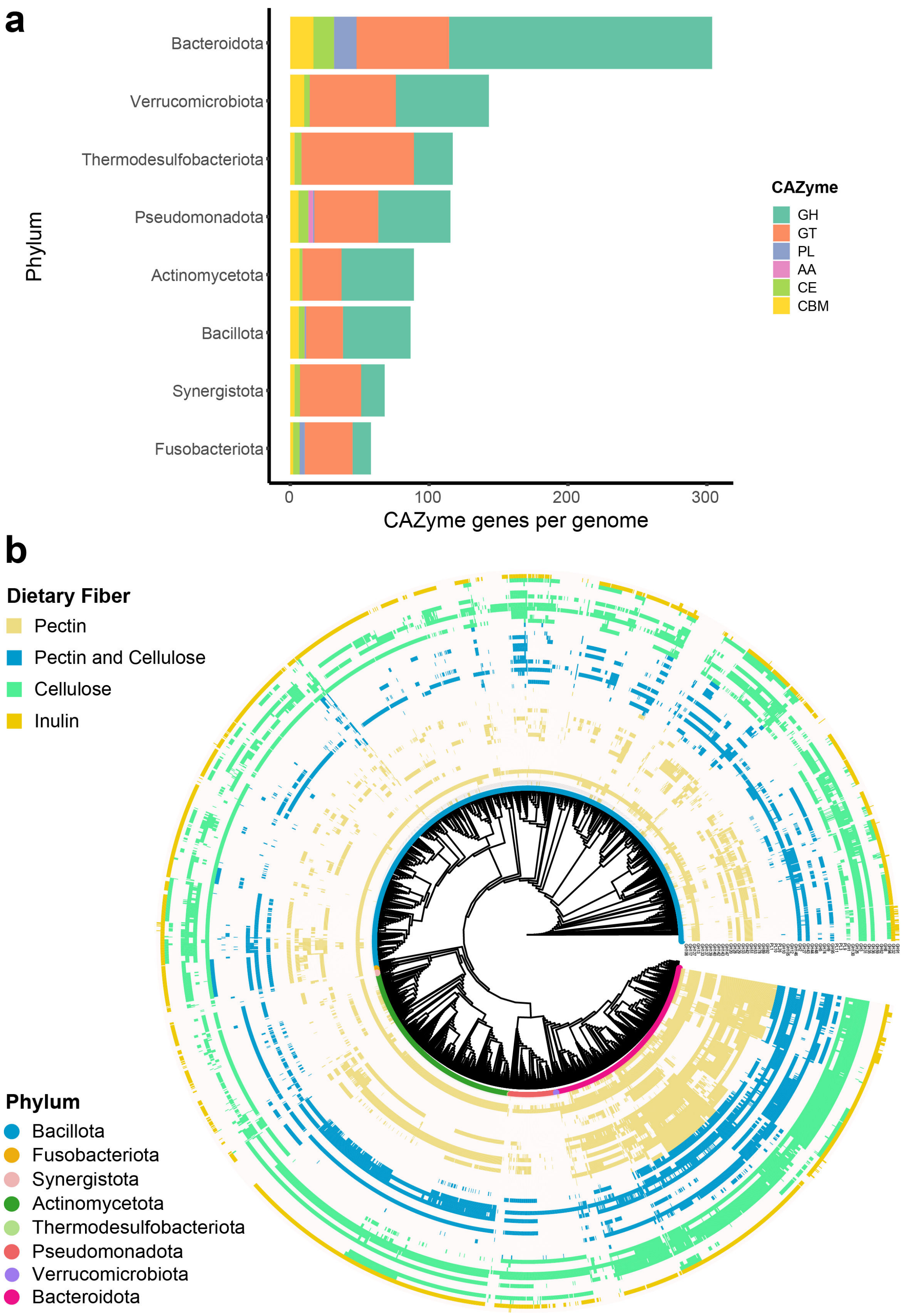

Supplementary Fig. 9: The distribution of CAZymes and dietary fiber-degrading CAZymes in CGR2 a, The average number of CAZyme genes in all genomes of each phylum is shown. CAZyme families are marked with different colors. **b,** Inulin-, cellulose-, and inulin-degrading GHs and PLs collected from the dbCAN-PUL databases. The presence or absence of dietary fiber-degrading GH and PL genes in each genome of CGR2 is shown.

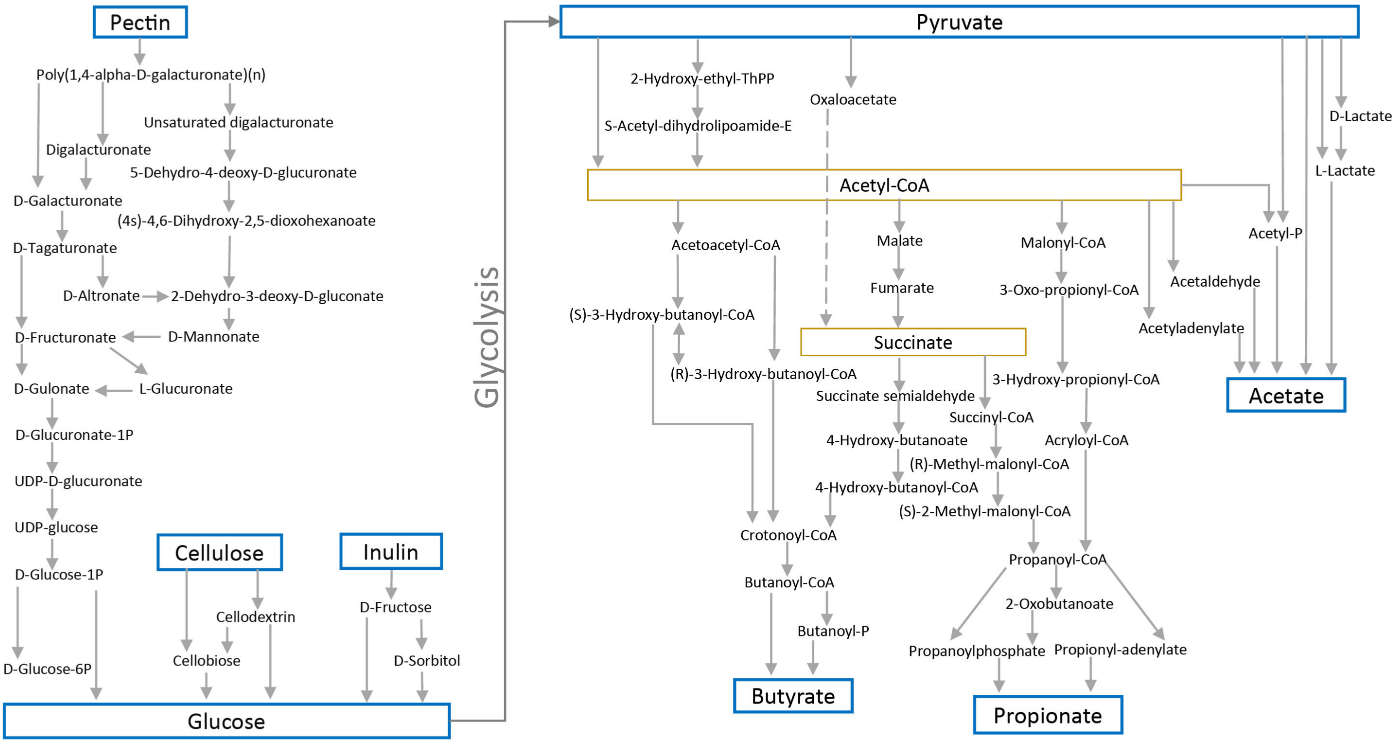

Supplementary Fig. 10: Detailed pathways involved in the degradation of cellulose, inulin, and pectin, and pathways involved in the synthesis of acetate, propionate and butyrate.

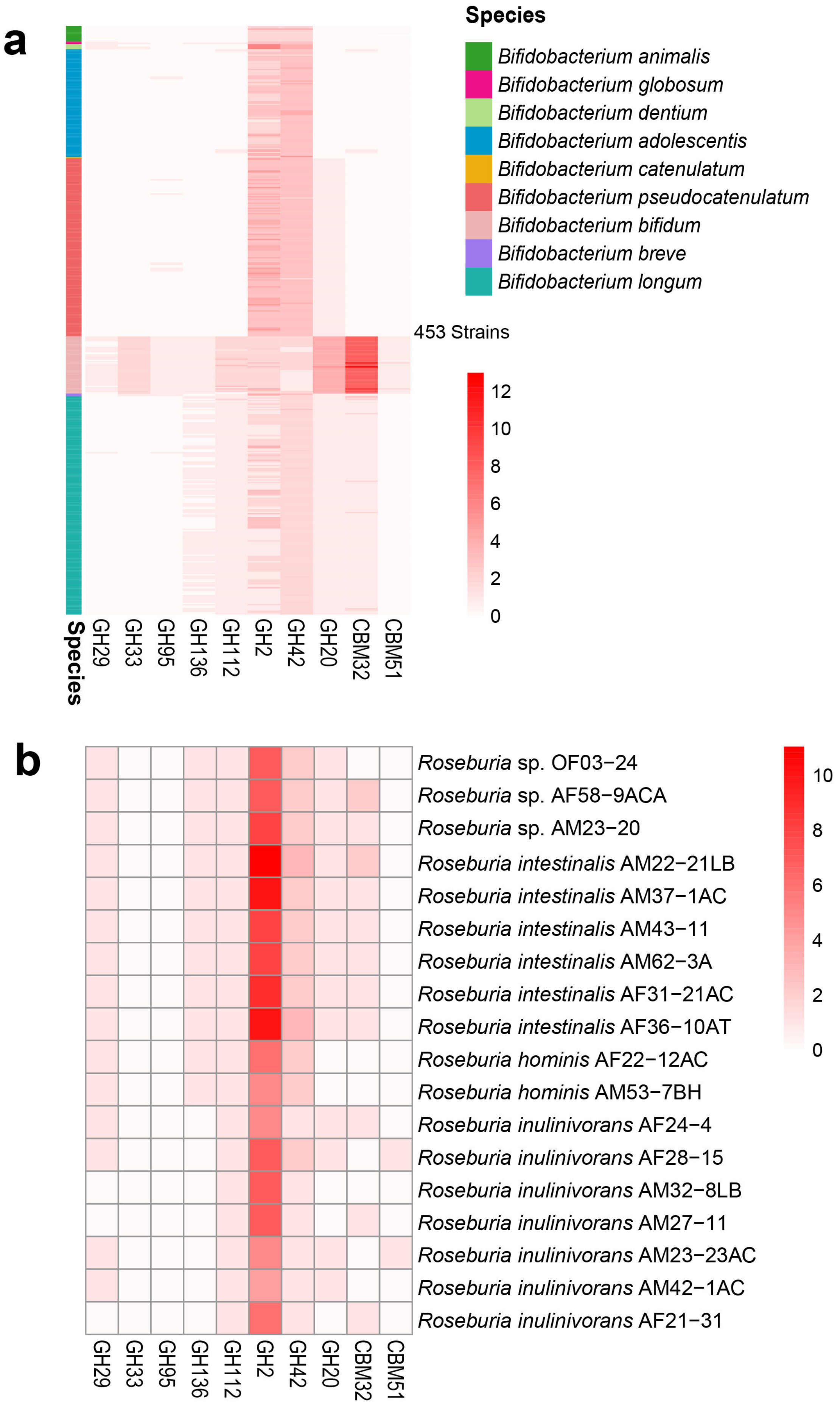

Supplementary Fig. 11: Distribution of HMO-degrading CAZymes in bifidobacteria and *Roseburia*. a, The number of HMO-degrading CAZyme genes in each bifidobacteria genome. **b,** The number of HMO-degrading CAZyme genes in each *Roseburia* genome.

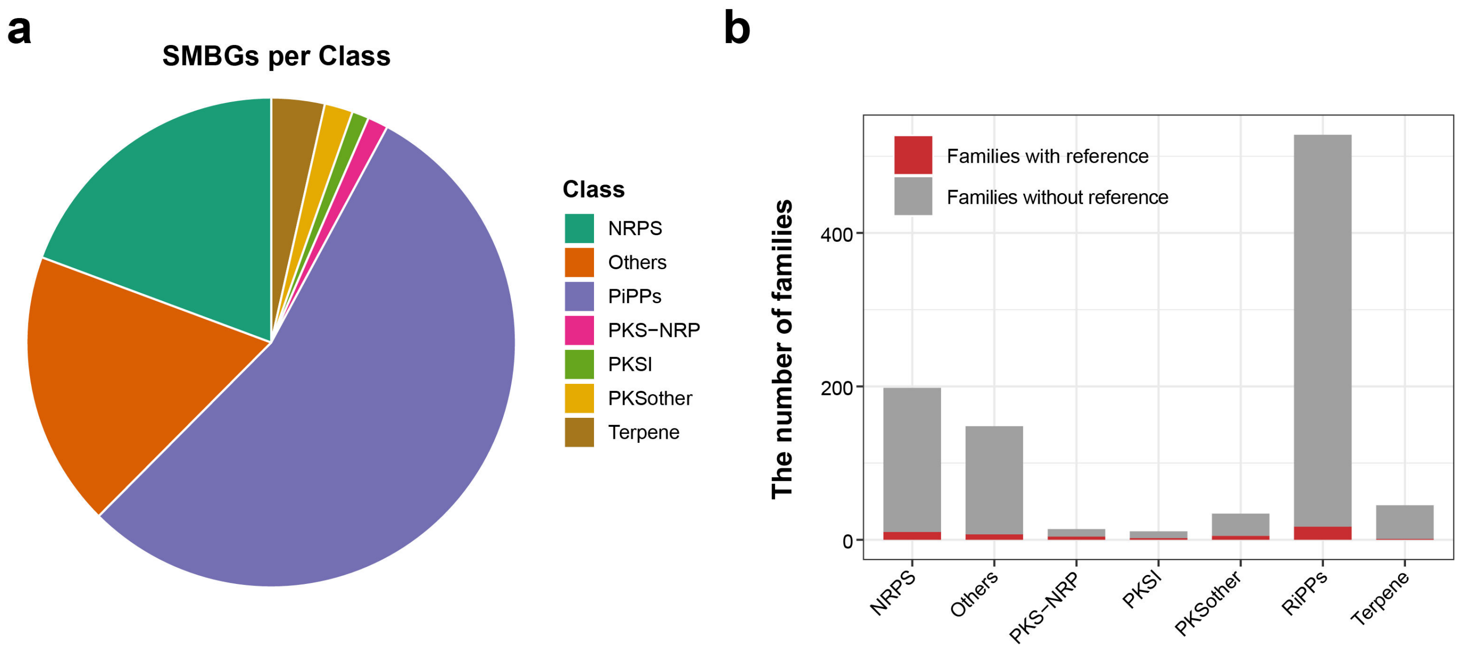

Supplementary Fig. 12: Clustering SMBGs into Classes. a, The proportion of each SMBGs class. **b,** Statistics of the number of references contained in each SMBGs classes.

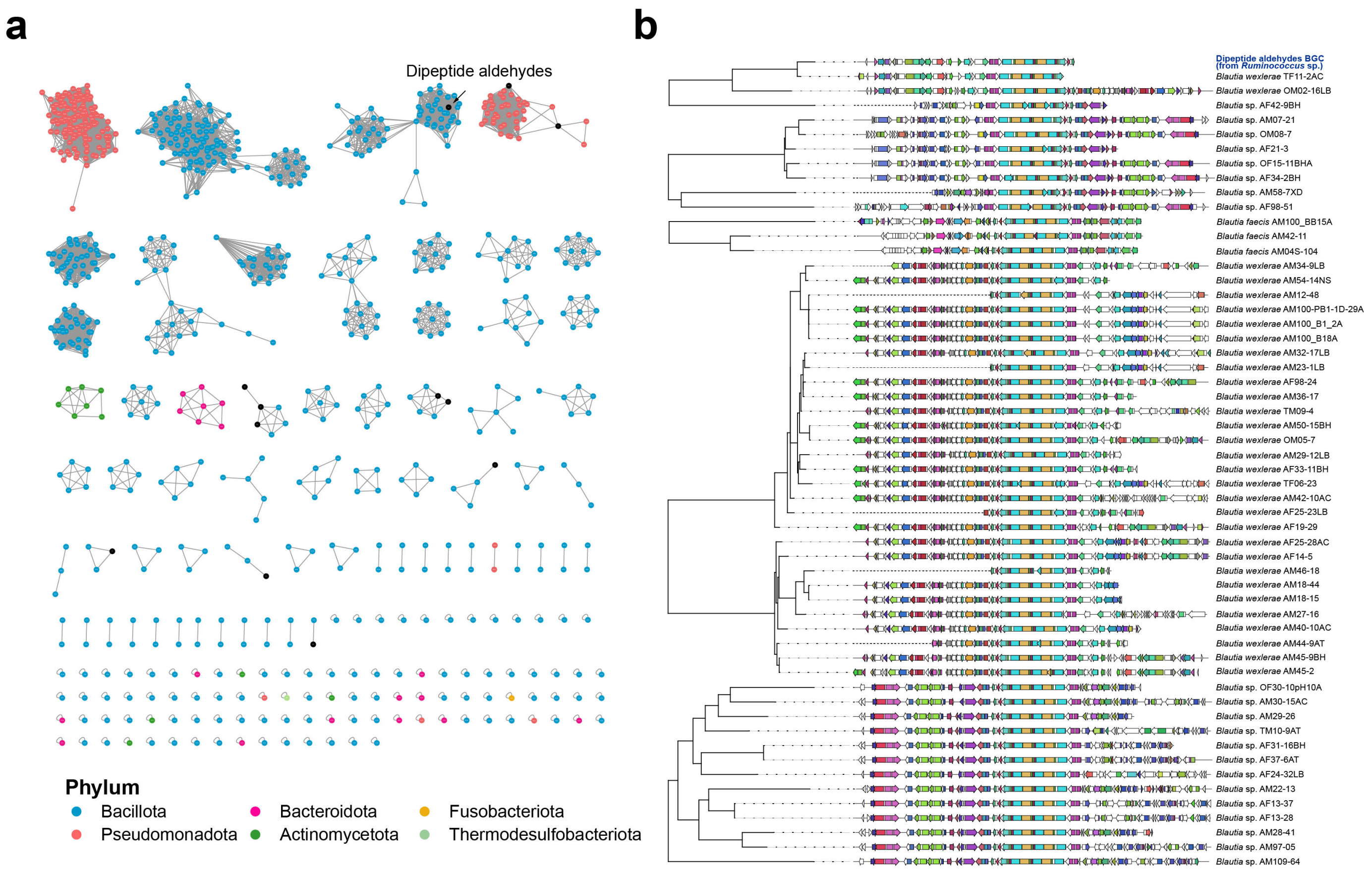

Supplementary Fig. 13: Analysis of the NRP classes. a, Sequence similarity network of NRPs. **b,** Sequence evolution relationship of dipeptide aldehydes.

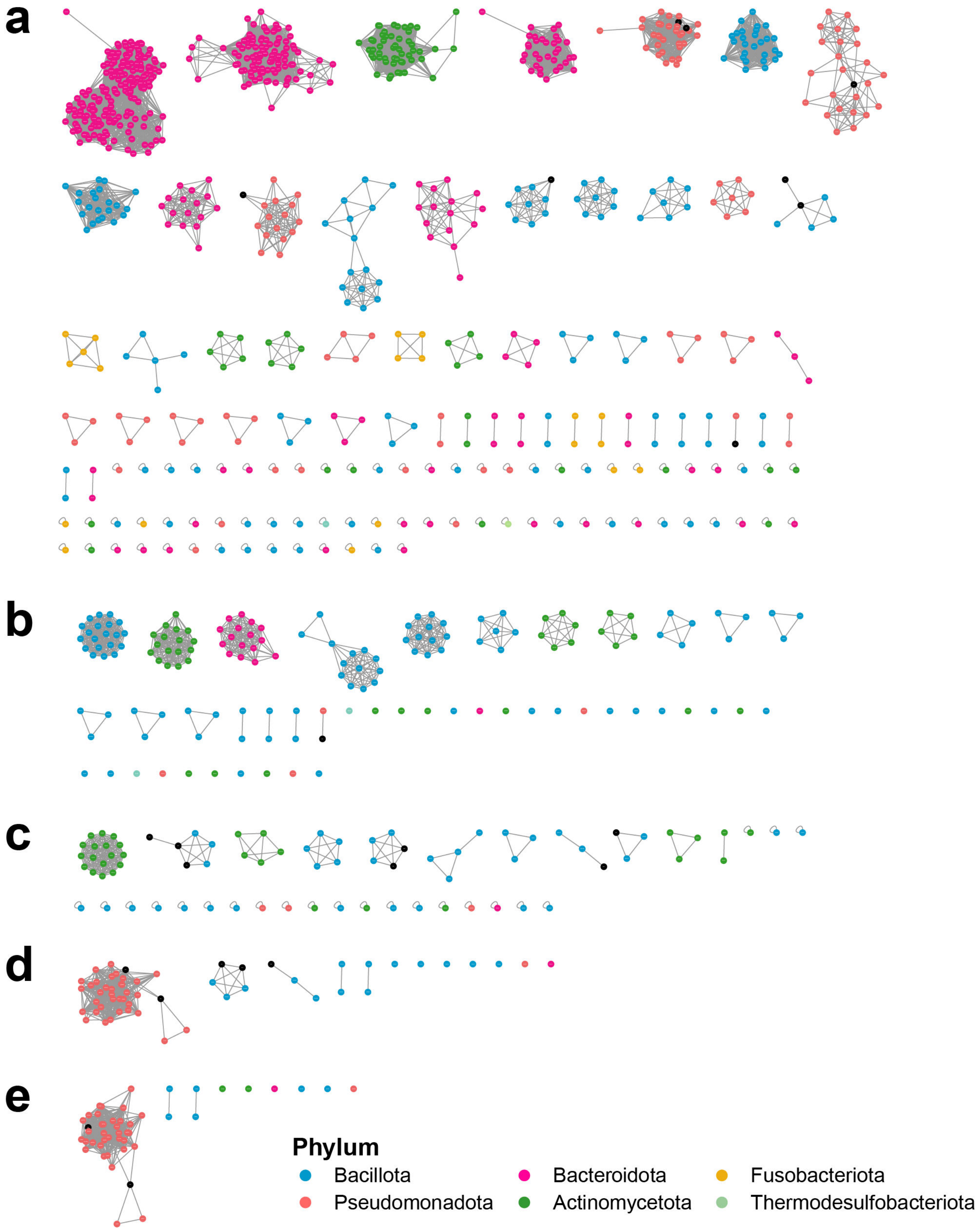

Supplementary Fig. 14: Analysis of sequence similarity networks. Others (**a**), Terpene (**b**), PKS-other (**c**), PKS-NRP (**d**) and PKSI (**e**).

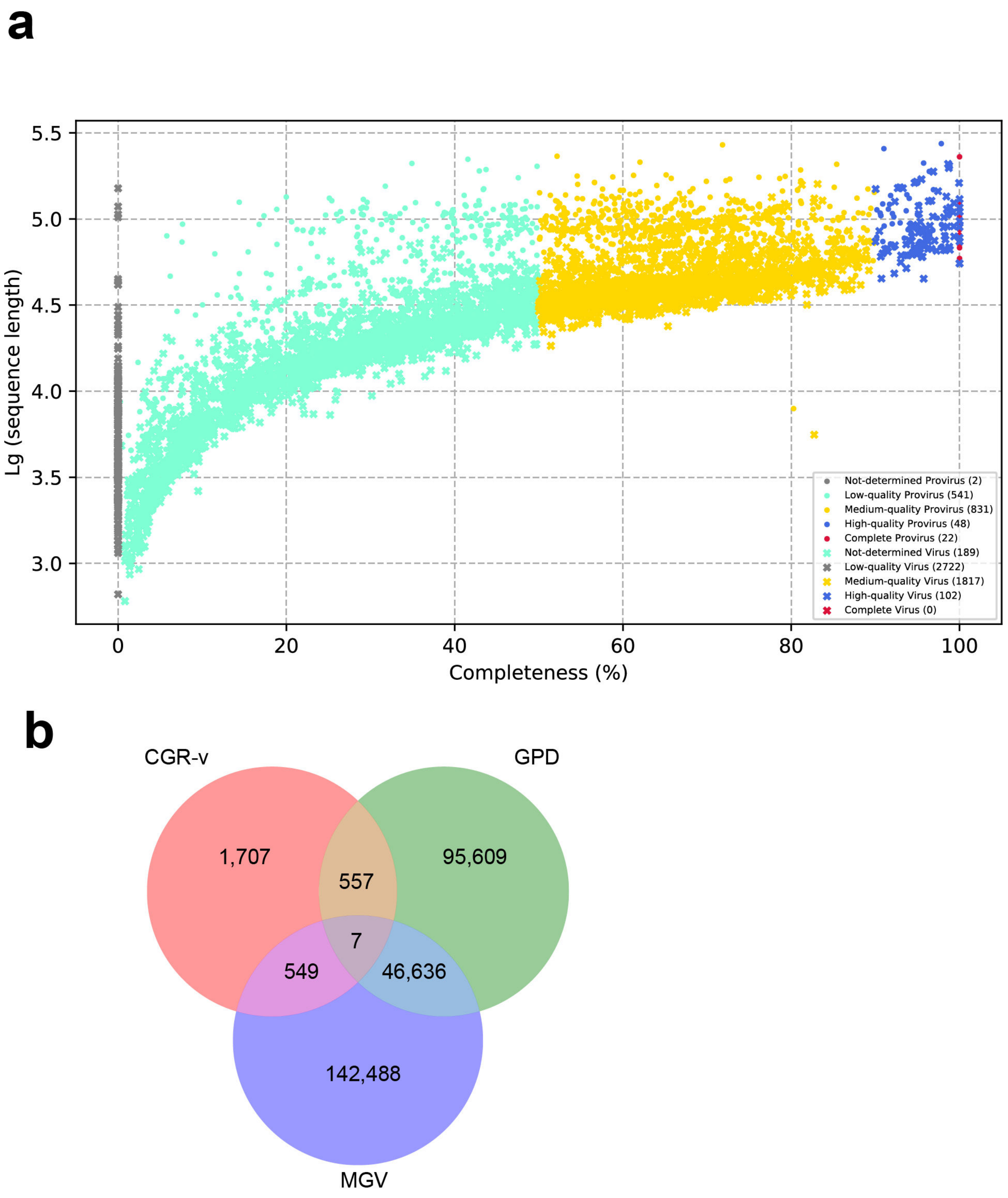

Supplementary Fig. 15: Characteristics of CGR-v. a, CheckV assessment of viral sequences. Quality ratings are shown in different colors, while two shapes mean different virus types. **b,** Comparison with MGV and GPD.

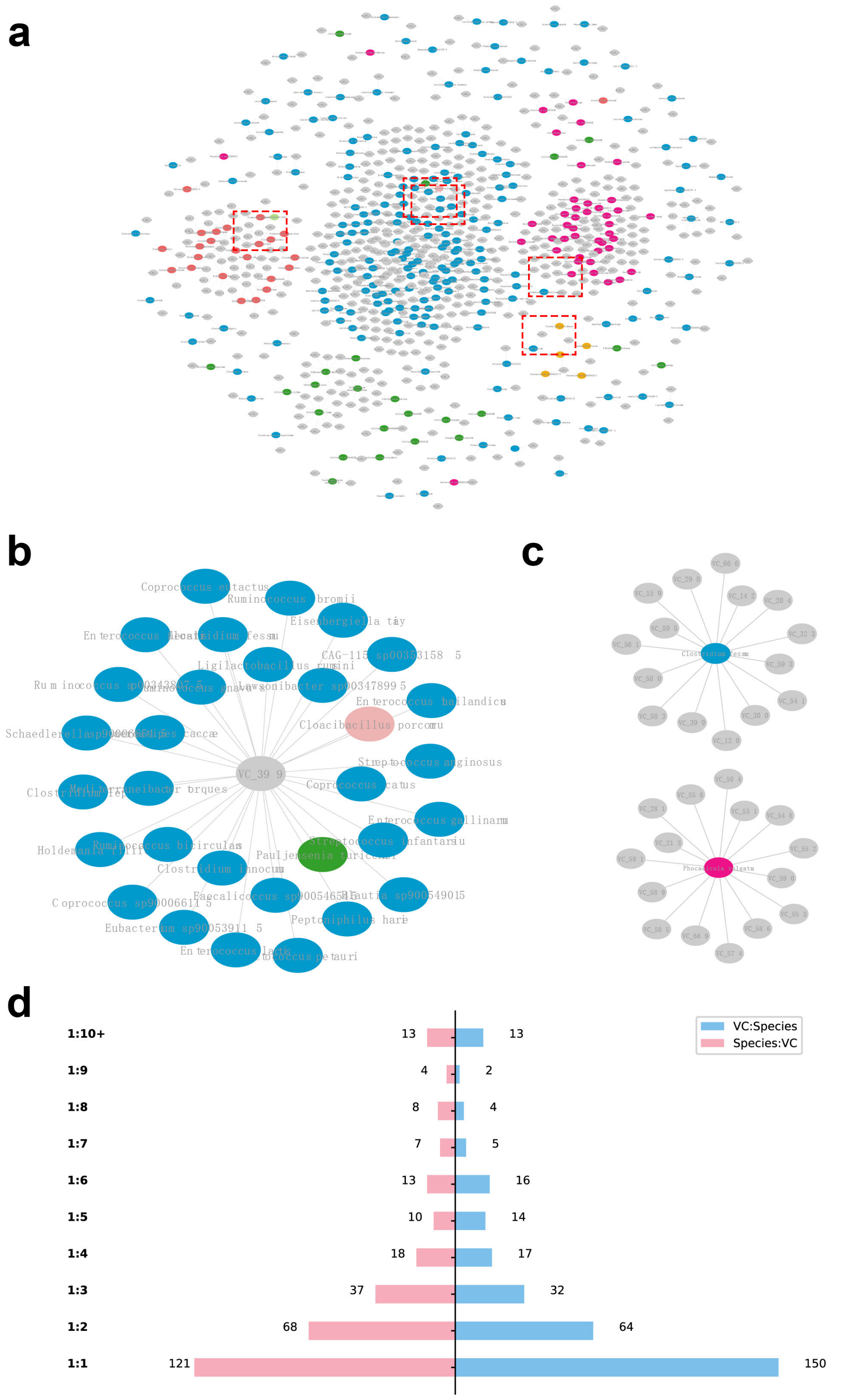

Supplementary Fig. 16: Infection network of phages-bacteria. a, Network diagram of infection relationship. Grey nodes represent VCs, colored nodes represent bacterial species of different phyla. **b,** Host range of the most infectious VC in CGRv, VC_399. **c,** The most infected species: *Clostridium fessum* and *Phocaeicola vulgatus*. **d,** Quantification of the host range. The pink bars show the number of bacteria that one viral cluster can infect. The blue bars show the number of viral clusters that one bacterial species can host.

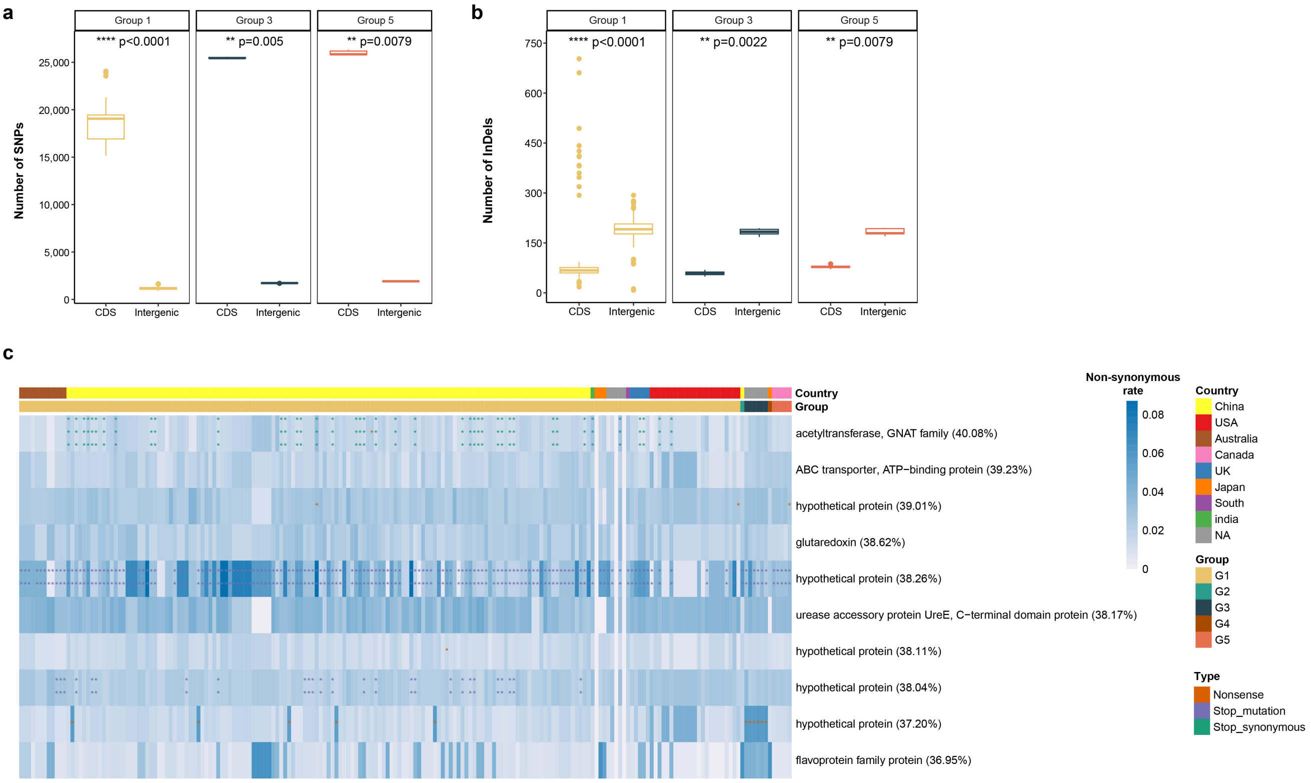

Supplementary Fig. 17: Variation statistics in CDS regions and intergenic regions for each group. a-b, SNP (**a**), and InDel (**b**). Boxplots show median, 25th and 75th percentile, the whiskers indicate the minima and maxima, and the points laying outside the whiskers of boxplots represent the outliers. Colors represent the group. *P* values were calculated using Wilcoxon rank-sum test (two-sided) (Group 1: *n*=184, Group 3: *n*=6, Group 5: *n*=5, *n* values refer to the number of independent results used to derive statistics). **c**, Variation of the top 10 CDSs. The heatmap is colored according to the non-synonymous mutation rate (Non-synonymous mutation frequency / CDS length), and “*” indicates mutation types. Synonymous mutations are not shown in the figure.

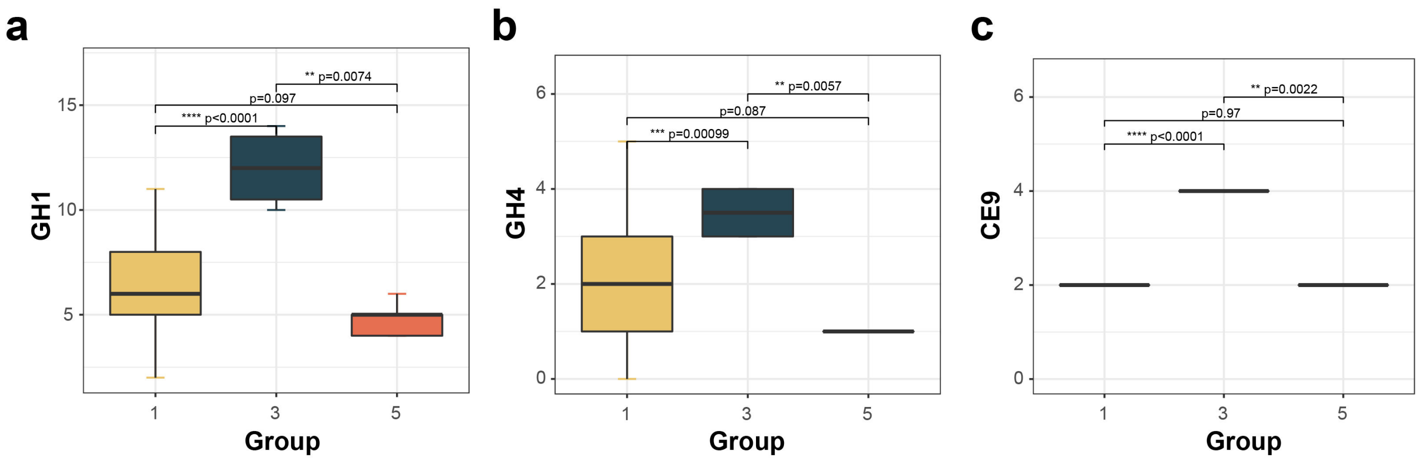

Supplementary Fig. 18: *Collinsella aerofaciens* genomic comparison of 130 CGR2 isolate-based genomes and 67 public genomes. (**a-c**) Differences in gene copy number of CAZymes GH1 (**a**), CH4 (**b**) and CE9 (**c**) between groups. Boxplots show median, 25th and 75th percentile, the whiskers indicate the minima and maxima, and the points laying outside the whiskers of boxplots represent the outliers. Colors represent the group. *P* values were calculated using Wilcoxon rank-sum test (two-sided) (Group 1: *n*=184, Group 3: *n*=6, Group 5: *n*=5, *n* values refer to the number of independent results used to derive statistics).
