# Supplementary material for: The genomic landscape of reference genomes of cultivated human gut bacteria
Source: Nat Commun. 2023 Mar 25;14:1663. doi: 10.1038/s41467-023-37396-x (PMC10039858; doi:10.1038/s41467-023-37396-x)
Supplement: Supplementary file 3 — Description of Additional Supplementary Files [file 41467_2023_37396_MOESM3_ESM.docx]

**Description of Additional Supplementary Files**

**File Name:** Supplementary Data 1

**Description:** Statistics for sequencing data of the 3,324 high-quality genomes.

**File Name:** Supplementary Data 2

**Description:** Taxonomic information of the 3,324 high-quality genomes.

**File Name:** Supplementary Data 3

**Description:** Metagenomics profiles of representative clusters in cohorts of healthy individuals from China, the Netherlands, and HMP. For the 179 previously unidentified species sheet, *P* values were calculated using Wilcoxon rank-sum test (two-sided) (China: n=3,550, HMP: n=661, the Netherlands: n=6,366, n values refer to the number of independent results used to derive statistics)

**File Name:** Supplementary Data 4

**Description:** Unique genes in the CGR2 genomes compared with the mapped MAGs.

**File Name:** Supplementary Data 5

**Description:** Number of CAZyme genes in each genome of CGR2.

**File Name:** Supplementary Data 6

**Description:** Detailed information of pathways involved in glycan degradation and SCFA synthesis in CGR2.

**File Name:** Supplementary Data 7

**Description:** HMO-degrading CAZyme genes in each genome of Bifidobacterium and Roseburia.

**File Name:** Supplementary Data 8

**Description:** Types and numbers of SMBGs identified by anti-SMASH in CGR2 genomes.

**File Name:** Supplementary Data 9

**Description:** Quality summary of “most confident” and “likely” phages and prophages.

**File Name:** Supplementary Data 10

**Description:** VC status, taxonomic information, and host information of CGRv.

**File Name:** Supplementary Data 11

**Description:** Protein clusters and their annotation.
