# Supplementary material for: The genomic landscape of reference genomes of cultivated human gut bacteria
Source: Nat Commun. 2023 Mar 25;14:1663. doi: 10.1038/s41467-023-37396-x (PMC10039858; doi:10.1038/s41467-023-37396-x)
Supplement: Supplementary file 5 — Reporting Summary [file 41467_2023_37396_MOESM5_ESM.pdf]

nature portfolio

Corresponding author(s): Liang Xiao, Karsten Kristiansen and Yuanqiang Zou

Last updated by author(s): Mar 2, 2023

Reporting Summary

Nature Portfolio wishes to improve the reproducibility of the work that we publish. This form provides structure for consistency and transparency in reporting. For further information on Nature Portfolio policies, see our Editorial Policies and the Editorial Policy Checklist.

Statistics

For all statistical analyses, confirm that the following items are present in the figure legend, table legend, main text, or Methods section.

n/a	Confirmed	
☐	☒	The exact sample size (*n*) for each experimental group/condition, given as a discrete number and unit of measurement
☐	☒	A statement on whether measurements were taken from distinct samples or whether the same sample was measured repeatedly
☐	☒	The statistical test(s) used AND whether they are one- or two-sided<br>*Only common tests should be described solely by name; describe more complex techniques in the Methods section.*
☒	☐	A description of all covariates tested
☐	☒	A description of any assumptions or corrections, such as tests of normality and adjustment for multiple comparisons
☐	☒	A full description of the statistical parameters including central tendency (e.g. means) or other basic estimates (e.g. regression coefficient) AND variation (e.g. standard deviation) or associated estimates of uncertainty (e.g. confidence intervals)
☐	☒	For null hypothesis testing, the test statistic (e.g. *F*, *t*, *r*) with confidence intervals, effect sizes, degrees of freedom and *P* value noted<br>*Give P values as exact values whenever suitable.*
☒	☐	For Bayesian analysis, information on the choice of priors and Markov chain Monte Carlo settings
☒	☐	For hierarchical and complex designs, identification of the appropriate level for tests and full reporting of outcomes
☒	☐	Estimates of effect sizes (e.g. Cohen's *d*, Pearson's *r*), indicating how they were calculated

Our web collection on statistics for biologists contains articles on many of the points above.

Software and code

Policy information about availability of computer code

Data collection	No software was used.
Data analysis	GeneMarkS-2 v1.10; CheckM v1.1.2; Prokka 1.14.6; fastANI v1.32; GTDB-Tk v2.1.0; Kraken v2.1.2; Bracken v2.5; iTOL v6.1.1; dbCAN v2.0; anti-SMASH v4.2.0; BiG-SCAPE/CORASON tool v1.0.1; Cytoscape v3.8.2; VirSorter v1.0.5; CheckV v0.7.0; Prodigal v2.6.3; VConTACT2 v0.9.19; MAFFT v7.407; FastTree v2.1.3; Mummer v3.22; lastz v1.03.73; TreeBeST v1.9.2; BLAST v2.2.26

For manuscripts utilizing custom algorithms or software that are central to the research but not yet described in published literature, software must be made available to editors and reviewers. We strongly encourage code deposition in a community repository (e.g. GitHub). See the Nature Portfolio guidelines for submitting code & software for further information.

Data

Policy information about availability of data

All manuscripts must include a data availability statement. This statement should provide the following information, where applicable:

- Accession codes, unique identifiers, or web links for publicly available datasets
- A description of any restrictions on data availability
- For clinical datasets or third party data, please ensure that the statement adheres to our policy

The genome data generated in this study have been deposited into CNSA of CNGBdb with accession number CNP0000126 and CNP0001833, and NCBI under the projects PRJNA482748 and PRJNA903559. All the bacterial strains in CGR2 have been deposited in China National GeneBank (CNGB), a non-profit, public-service-oriented organization in China. The strain information, including taxonomy, donor, and culture conditions can be found and accessed through https://db.cngb.org/

codeplot/datasets/CGR2.
The databases used in this study include GTDB database Release 07-RS207 (https://data.gtdb.ecogenomic.org/releases/release207/), dbCAN-PUL (https://bcb.unl.edu/dbcan_pul/Webserver/static/DBCAN-PUL/), KEGG database (https://www.genome.jp/kegg/), MIBiG version 1.4 (https://mibig.secondarymetabolites.org/), checkv-db-v0.6(https://portal.nersc.gov/CheckV/), and Genebank (www.ncbi.nlm.nih.gov/genbank/);
The datasets used in this study include human gut metagenome sequencing data of a Chinese cohort (a part of 4D-SZ) (https://db.cngb.org/search/project/CNP0000426/), HMP (https://portal.hmpdacc.org/), the Netherlands cohort (https://ega-archive.org/studies/EGAS00001005027), UHGG (http://ftp.ebi.ac.uk/pub/databases/metagenomics/mgnify_genomes), BIO-ML (https://www.ncbi.nlm.nih.gov/bioproject/PRJNA544527), hGMB (https://www.ncbi.nlm.nih.gov/bioproject/PRJNA656402), MGV (https://portal.nersc.gov/MGV/), and GPD (https://ftp.ebi.ac.uk/pub/databases/metagenomics/genome_sets/gut_phage_database/).

Human research participants

Policy information about studies involving human research participants and Sex and Gender in Research.

Reporting on sex and gender	Sex / gender was not included in our study design and analysis.
Population characteristics	This study recruited 299 participants from China, including 4 infants (aged 0-1), 7 children (aged 1-10), 30 teenagers (aged 11-20), 194 adults (aged 21-50), and 64 elders (aged >50).
Recruitment	We publish recruitment information through posters, and participants sign up voluntarily. All participants were recruited in ShenZhen, China; Fecal samples were collected from 299 healthy donors not taking any drugs during the last months prior to sampling. No additional volunteer selection criteria.
Ethics oversight	The collection of the 299 samples was approved by the Institutional Review Board on Bioethics and Biosafety of BGI.

Note that full information on the approval of the study protocol must also be provided in the manuscript.

Field-specific reporting

Please select the one below that is the best fit for your research. If you are not sure, read the appropriate sections before making your selection.

☒ Life sciences ☐ Behavioural & social sciences ☐ Ecological, evolutionary & environmental sciences

For a reference copy of the document with all sections, see nature.com/documents/nr-reporting-summary-flat.pdf

Life sciences study design

All studies must disclose on these points even when the disclosure is negative.

Sample size	This study collected 299 feces, isolated ~20,000 bacterial isolates, and generated 3324 high quality genomes. No sample size calculation was performed, but the sample size and amount of generated sequence data are larger than currently published studies of similar nature.
Data exclusions	genomes with < 90% completeness or > 10% contamination were excluded. Quality controls used to exclude genomes were based on previously published criteria.
Replication	Genomic data are publicly available, so analyzes can be reproduced using the data and software described in the Methods.
Randomization	Randomization is applied when we select strains from the same cluster for sequencing based on a threshold of 98.7% identity of the 16S rRNA gene sequence.
Blinding	Blinding is not necessary for this study, because is not influenced by the subjective factors of the subjects or researchers.

Reporting for specific materials, systems and methods

We require information from authors about some types of materials, experimental systems and methods used in many studies. Here, indicate whether each material, system or method listed is relevant to your study. If you are not sure if a list item applies to your research, read the appropriate section before selecting a response.

Materials & experimental systems

n/a	Involved in the study
☒	☐ Antibodies
☒	☐ Eukaryotic cell lines
☒	☐ Palaeontology and archaeology
☒	☐ Animals and other organisms
☒	☐ Clinical data
☒	☐ Dual use research of concern

Methods

n/a	Involved in the study
☒	☐ ChIP-seq
☒	☐ Flow cytometry
☒	☐ MRI-based neuroimaging
